# Novel Putative Transposable Element Associated with the Subtype E5 Botulinum Toxin Gene Cluster of Neurotoxigenic *Clostridium butyricum* Type E Strains from China

**DOI:** 10.3390/ijms23020906

**Published:** 2022-01-14

**Authors:** Tao Li, Nianzhi Ning, Angelo Iacobino, Liangyan Zhang, Hui Wang, Giovanna Franciosa

**Affiliations:** 1 State Key Laboratory of Pathogens and Biosecurity, Beijing Institute of Microbiology and Epidemiology, Beijing 100071, China; litaobmi@126.com (T.L.); ningnianzhi@163.com (N.N.); polini@live.cn (L.Z.); 2 Department of Infectious Diseases, Istituto Superiore di Sanità, 00161 Rome, Italy; angelo.iacobino@iss.it; 3 National Center for the Control and Evaluation of Medicines, Istituto Superiore di Sanità, 00161 Rome, Italy

**Keywords:** *Clostridium butyricum*, *Clostridium botulinum*, whole genome sequence, botulinum neurotoxin gene cluster, transposable elements

## Abstract

Previously, a whole-genome comparison of three *Clostridium butyricum* type E strains from Italy and the United States with different *C. botulinum* type E strains indicated that the *bont*/*e* gene might be transferred between the two clostridia species through transposition. However, transposable elements (TEs) have never been identified close to the *bont*/*e* gene. Herein, we report the whole genome sequences for four neurotoxigenic *C. butyricum* type E strains that originated in China. An analysis of the obtained genome sequences revealed the presence of a novel putative TE upstream of the *bont*/*e* gene in the genome of all four strains. Two strains of environmental origin possessed an additional copy of the putative TE in their megaplasmid. Similar putative TEs were found in the megaplasmids and, less frequently, in the chromosomes of several *C. butyricum* strains, of which two were neurotoxigenic *C. butyricum* type E strains, and in the chromosome of a single *C. botulinum* type E strain. We speculate that the putative TE might potentially transpose the *bont*/*e* gene at the intracellular and inter-cellular levels. However, the occasional TE occurrence in the clostridia genomes might reflect rare transposition events.

## 1. Introduction

Botulism is a disease that affects humans, other mammals, birds and fish. It results from the blockade of neurotransmitter release at the peripheral junctions by the botulinum neurotoxin (BoNT). Of the nine distinct BoNT serotypes that have been identified so far (BoNT/A through H and BoNT/X), BoNT/E is among those that more frequently cause botulism in humans [1].

BoNT/E can be produced by the *Clostridium botulinum* strains of phylogenetic Group II and atypical strains of neurotoxigenic *C. butyricum* (phylogenetic Group VI). Clostridia phylogenetic Groups (I through VI) can be considered separate species [2,3].

In Italy, where neurotoxigenic *C. butyricum* type E strains were originally isolated, those microorganisms have been involved in type E botulism more frequently than *C. botulinum* strains. Indeed, they have recurrently been recovered from cases of infant and adult intestinal toxemia botulism (a form of botulism resulting from the absorption of BoNT released in the gut by colonizing neurotoxigenic clostridia) and from a suspected case of foodborne botulism due to the ingestion of BoNT in contaminated food [4,5,6,7].

In China, where type E foodborne botulism shows different features from that in other countries because it is more often related to the consumption of contaminated fermented beans and grains rather than contaminated seafood products, as is most frequently the case elsewhere [8], *C. butyricum* type E strains have been isolated from several outbreaks of foodborne botulism in the 1990s, as well as from the environment [9,10]. It is intriguing that during a more recent investigation of a case of infant botulism in the United Kingdom due to *C. butyricum* type E infection associated with environmental contamination by pet turtles, the microorganism was also recovered from an open container of turtle feed originally sourced in China [11].

Early genetic studies divided the clinical *C. butyricum* type E strains isolated in Italy and those recovered in China from clinical and non-clinical (food and environmental) sources into three distinct clonal groups; all strains were shown to carry the *bont*/*e* gene in their chromosome and possessed a megaplasmid of sizes ranging from 600 to 800 kb [12,13].

Moreover, based on the *bont*/*e* gene sequences, the Italian and Chinese *C. butyricum* type E strains were predicted to produce two different BoNT/E subtypes, E4 and E5, respectively, differing from each other by 5.1% in amino acid composition [14,15], which is within the 2.6% cut-off value of the amino acid difference required for defining BoNT subtypes [16].

Currently, only three genome sequences are available for as many BoNT/E4-producing *C. butyricum* strains isolated from infant botulism cases; two in Italy and one in the United States [17,18,19,20]. The genome sequence of the US strain is complete and consists of a chromosome bearing the *bont*/*e* gene and two plasmids of 800 and 9 kb [19].

In all three genomes, the *bont*/*e* gene is located at the 3′ end of a conserved gene cluster that is flanked by incomplete insertion sequences (ISs’) [17,18,19]. A transposon-associated resolvase (*rarA*) gene is present downstream of the *bont*/*e* gene cluster and the whole genetic region, including the *rarA* gene, is inserted into a split *rarA* gene of a genetic lineage different from that of its intact counterpart [17,20]. Similar *bont*/*e* gene cluster organization and location in a split chromosomal *rarA* operon have been observed in several *C. botulinum* type E strains’ genomes, which suggests that the *rarA* gene is the target of a possible *bont*/*e* gene cluster transposition in different *C. butyricum* and *C. botulinum* species [15,17,20].

The purpose of this study was to obtain and compare the whole genome sequences (WGSs) of four Chinese BoNT/E5-producing *C. butyricum* strains from foodborne botulism outbreaks and environmental sources to better define the genetic diversity in Group VI *C. butyricum* type E strains and to characterize the composition and organization of their subtype E5 botulinum toxin gene clusters and adjacent regions.

Our results show for the first time the presence of a putative transposable element (TE) at the 3′ end of the *bont*/*e5* gene clusters.

## 2. Results

### 2.1. C. butyricum Type E Strains and General Genome Features

Strains LCL 063 and LCL 155 were originally isolated from a clinical sample and fermented soybean paste, respectively, during investigations of distinct foodborne botulism outbreaks in China [9,10,12]. Strains KZ 1886 and KZ 1890 were recovered from soil samples collected from the Weishan Lake area, where type E foodborne botulism outbreaks had occurred [12].

The general features of the WGSs of the four neurotoxigenic *C. butyricum* type E are summarized in Table 1.

The genomes consisted of 4,523,830 to 4,633,810 bp, depending on the strain, with an average GC content of 28.7%, consistent with the low GC content reported for the genus Clostridium [21]. In the genome of the four strains, 4043 to 4232 CDS were identified.

Basic Local Alignment Search Tool (BLAST) analyses revealed that the 16S rRNA genes of the four isolates showed 99% identity with those of strains of the *C. butyricum* species, thus confirming that they belong to the *C. butyricum* species.

The presence of a megaplasmid in each genome sequence was inferred by comparison with the available completed genome sequence of strain CDC 51208, which includes an 820 kb plasmid (pNPD4_2, accession No. CP013238.1). By contrast, the genome sequences generated in this study did not contain any regions showing similarity to the other 9 kb plasmid (pNPD4_1, accession No. CP13240.1) identified in the *C. butyricum* type E strain CDC 51208 [19], which suggests that the four Chinese *C. butyricum* type E strains likely do not contain a similar smaller plasmid.

The *bont*/*e* gene was confirmed to be located in the chromosomes of strains, as previously indicated by pulsed field gel electrophoresis (PFGE) and Southern blot analyses with specific *bont*/*e* gene probes [12,13].

By using the PHAge Search Tool Enhanced Release web server (PHASTER, http://phaster.ca/, accessed on 5 December 2021), the presence of two intact prophages was predicted in all strains, except for strain KZ 1890. Specifically, strain KZ 1886 contained two prophages that exhibited homology with phages infecting *C. difficile* and *Lactococcus*. Strains LCL 063 and LCL 155 shared identical prophages that matched the *C. difficile* phage PhiCD6356 and *Paenibacillus* virus Lily. Different scaffolds harbored either the prophages or *bont*/*e* locus regions, indicating that the prophages and *bont*/*e* loci were not physically associated in the three genome sequences.

In addition, the genome annotation by Prokka1.14.6 revealed an *enterotoxin* gene in each genome sequence generated in this study. The *enterotoxin* genes showed 100% coverage and 98% to 99% identity with an *enterotoxin* gene previously identified in the genome sequence of a non-neurotoxigenic *C. butyricum* strain isolated from a patient with diarrhea [22]. A BLASTN search using this gene sequence as a query revealed a similar *enterotoxin* gene in the genome sequences of several *C. butyricum* strains, including the neurotoxigenic *C. butyricum* type E4 strains BL 5262 and Cbu 5521 from Italy and CDC 51208 from the United States, as well as some non-neurotoxigenic *C. butyricum* strains isolated from infants with necrotizing enterocolitis [23]. Whether this enterotoxin is produced by the neurotoxigenic *C. butyricum* type E strains and plays a pathogenic role remains to be determined.

### 2.2. Comparative Genomic Analysis

To evaluate the phylogenetic relationships in the four neurotoxigenic *C. butyricum* type E strains (LCL 063, LCL 155, KZ 1886 and KZ 1890) and to examine their evolutionary pathway, a phylogenetic tree was built by including in the analysis the genome sequences available in GenBank from three other strains of neurotoxigenic *C. butyricum* type E (CDC 51208, BL 5262, and Cbu 5521), four non-neurotoxigenic *C. butyricum* type E strains (KNU-L09, CBUT, CFSA3987 and CFSA3989), and a *C. botulinum* type E strain (Alaska E43). The latter strains were used as references for the respective clostridia species.

**Figure 1:**
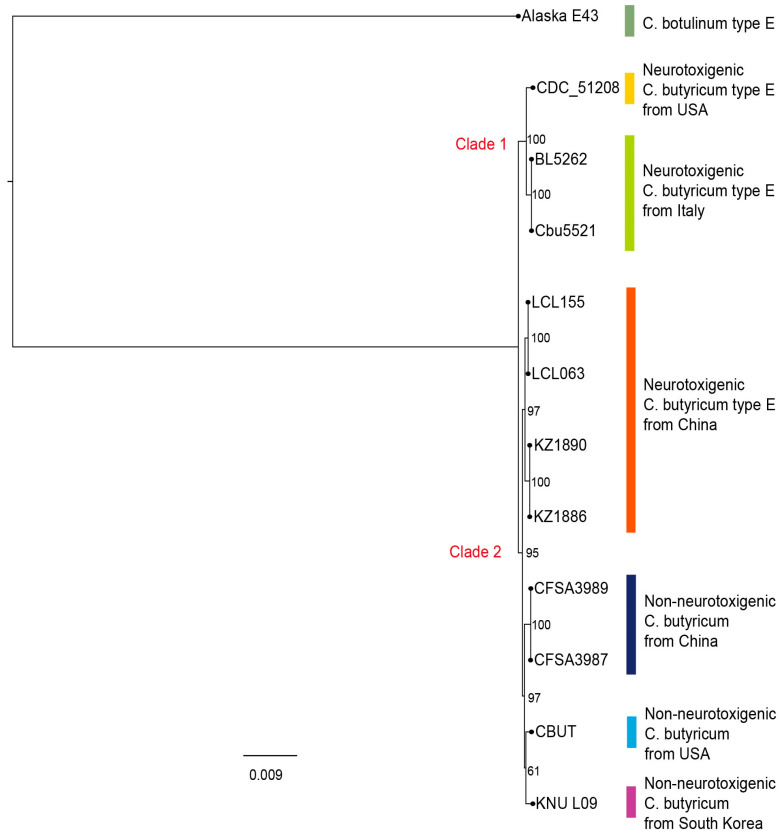
A phylogenetic tree was built by including one strain of *C. botulinum* type E (Alaska E43), one strain of non-neurotoxigenic *C. botulinum* type E (KNU-L09), and seven strains of neurotoxigenic *C. butyricum* type E (CDC51208, Cbu5521, BL5262, LCL-063, LCL-155, KZ 1886 and KZ 1890). A maximum-likelihood phylogenetic tree was constructed using the single-nucleotide polymorphisms identified between strains by kSNP3.

All *C. butyricum* strains clustered together and were grouped into two major clades (Figure 1), one of which (clade 1) included the genomes of the neurotoxigenic *C. butyricum* type E strains from Italy and the United States while the other (clade 2) consisted of the genomes of the four *C. butyricum* type E strains from China determined in this study and the non-neurotoxigenic *C. butyricum* strains. The analysis also confirmed that the clinical and food strains (LCL 063 and LCL 155) were closely related but not identical to the soil strains (KZ 1886 and KZ 1890) [12,13]. As expected, the *C. botulinum* type E strain Alaska E43 clustered distantly (Figure 1).

Thus, the phylogenetic analysis of the genome sequences revealed that the four neurotoxigenic *C. butyricum* type E strains from China substantially differed from the *C. butyricum* type E strains isolated in Italy—as was previously determined by PFGE analyses [12,13]—and from the strain from the United States; by contrast, a closer relationship was found between the four Chinese strain genomes and the genome of non-neurotoxigenic *C. butyricum* strains from different geographic areas.

### 2.3. Comparative Analyses of the Bont/e Gene Clusters and Adjacent Regions

**Figure 2:**
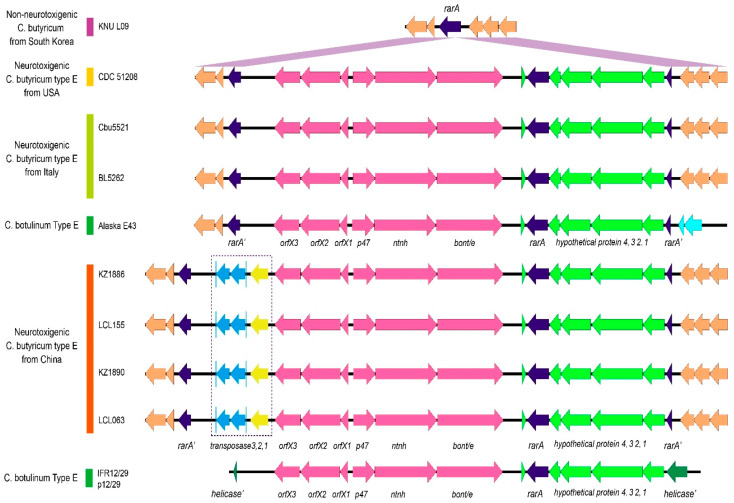
Gene arrangement of the DNA cassette containing the *bont*/*e* gene cluster. The *bont*/*e* gene cluster and flanking regions are indicated for the different strains. Three *transposase* genes are only present in the four Chinese strains (LCL063, LCL155, KZ 1886 and KZ 1890).

The gene organization and structure of the *bont*/*e* gene clusters and flanking regions in the four Chinese neurotoxigenic *C. butyricum* type E strains (LCL 063, LCL 155, KZ 1886 and KZ 1890) are presented in Figure 2.

The ~13 kb *bont*/*e* gene clusters were identical in content and arrangement to those in the genome of other BoNT/E-producing clostridia, consisting of the six genes (5′ to 3′) *orfX3*-*orfX2*-*orfX1* and, in the opposite direction, *p47*-*ntnh*-*bont*/*e*.

The neurotoxin gene clusters of BoNT/E-producing *C. botulinum* and *C. butyricum* strains have been reported to be part of a large gene cassette of ~24 kb that includes six additional conserved genes downstream of the *bont*/*e* gene, of which one is an intact *rarA* gene and the remaining ones are hypothetical genes; this 24 kb gene cassette may be inserted into either a chromosomal *rarA* gene or a plasmid *helicase* gene depending on the strain (Figure 2) [17,20].

Accordingly, in the genomes of the four Chinese *C. butyricum* type E strains in this study, a chromosomal *rarA* gene was found to be interrupted by an insert region containing the *bont*/*e* gene cluster. However, surprisingly, the inserted cassette region was 27 kb (precisely, 26,848 bp) rather than 24 kb, as previously determined in other BoNT/E-producing *C. botulinum* and *C. butyricum* strains (Figure 2). The nucleotide sequences of the ~27 kb insert regions carrying the *bont*/*e* neurotoxin gene cluster were 100% identical in the four Chinese *C. butyricum* type E strains.

**Figure 3:**
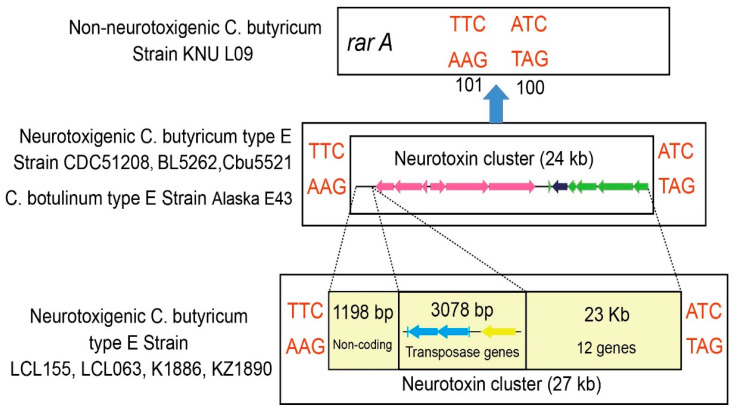
DNA insertion site for the 24 or 27 kb DNA cassette containing the *bont*/*e* gene cluster. The *bont*/*e* gene cluster is inserted into a *rarA* gene in different neurotoxigenic *C. butyricum* type E strains. An additional 3078 bp sequence is located immediately upstream of the *bont*/*e* gene cluster in the four Chinese *C. butyricum* type E strains (LCL 063, LCL 155, KZ 1886 and KZ 1890).

The analysis of this ~27 kb insert region revealed that it was similar in gene content and arrangement to the ~24 kb insert region described in other BoNT/E-producing clostridia strains [17,20], except for the presence of three additional genes encoding as many different transposases located immediately upstream of the *bont*/*e* gene cluster, which is 377-bp bases from the *orfX3* gene and accounted for 3087 bp (Figure 2 and Figure 3). Detailed descriptions of the three *transposase* genes will be provided later in the text.

Using the entire 26,848 bp insert region, related sequences were searched through a BLAST analysis against the database of completely sequenced microbial genomes. The results indicated an 88% coverage rate with 12 completed genomes of BoNT/E-producing strains, including nine *C. botulinum* type E strains and three strains of neurotoxigenic *C. butyricum* type E (CDC 51208, BL 5262 and Cbu 5521); the sequence encompassing three *transposase* genes was absent from all covered similarity regions. The most closely related sequence, with a 99.67% identity rate, was found in the plasmid (p12/29) of the *C. botulinum* type E strain IFR 12/29. A slightly lower identity rate (98.95%) was found in the homologous region in the chromosome of the *C. butyricum* type E strain CDC 51208, which is nearly identical (coverage 100%, 23760/23761 nucleotide identity) to both homologous regions in the assembled genomes of the Italian *C. butyricum* type E strains BL 5262 and Cbu 5521.

**Figure 4:**
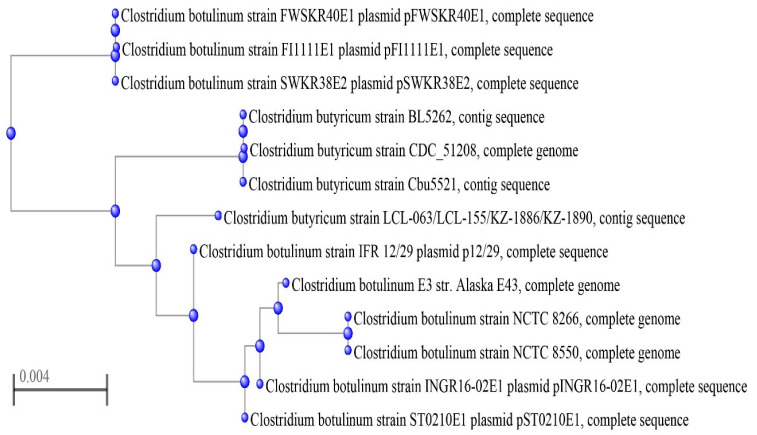
Fast minimum evolution trees of the 24 or 27 kb DNA cassette containing the *bont*/*e* gene cluster. The analysis included nine strains of *C. botulinum* type E (FWSKR40E1, FI1111E1, SWKR38E2, IFR12/29, Alaska E43, NCTC 8266, NCTC 8550, INGR16-02E1 and ST0210E1) and seven strains of *C. butyricum* type E (CDC 51208, Cbu 5521, BL 5262, LCL 063, LCL 155, KZ 1886 and KZ 1890).

A fast minimum evolution tree [24] of the insert region sequences from the four Chinese *C. butyricum* type E strains and additional BoNT/E-producing clostridia is presented in Figure 4.

#### 2.3.1. *Bont*/*e* Genes

The *bont*/*e* gene (3756 bp) sequences obtained in this study for the Chinese strains (LCL 063, LCL 155, KZ 1886 and KZ 1890) were analyzed using BLAST by query against the nucleotide collection (nr/nt) database of the National Center for Biotechnology Information (NCBI). They were found to be 100% identical to the *bont*/*e5* gene sequences determined previously for the same *C. butyricum* type E strains [12].

Moreover, the analysis revealed a high coverage (>99%) with 51 sequences, of which 48 were from *C. botulinum* type E strains and the remaining three were from *C. butyricum* type E strains (CDC 51208, BL 5256 and Cbu 5521) (Figure S1, Supplementary Materials). The highest identity rate (98.30%) was with the *bont*/*e1* gene sequences of some *C. botulinum* strains, including those in the plasmids of *C. botulinum* strains IFR 12/29 and CB 11/1-1. The identity rate with the *bont*/*e4* genes of the *C. butyricum* strains (CDC 51208, BL 5256 and Cbu 5521) was 97.29%.

#### 2.3.2. *RarA* Genes

Similarly to other BoNT/E-producing *C. butyricum* and *C. botulinum* strains, the *C. butyricum* type E strains in this study were found to contain two copies of the *rarA* gene, of which one was split by the insertion of the ~27 kb insert cassette containing the *bont*/*e* gene cluster, other genes, and another intact *rarA* gene (Figure 2). The intact (1245 bp) *rarA* gene and spliced (reconstructed) (1242 bp) *rarA* gene sequences of the Chinese strains were quite different from each other, showing only 905/1242 nucleotide identity (72.87%).

**Figure 5:**
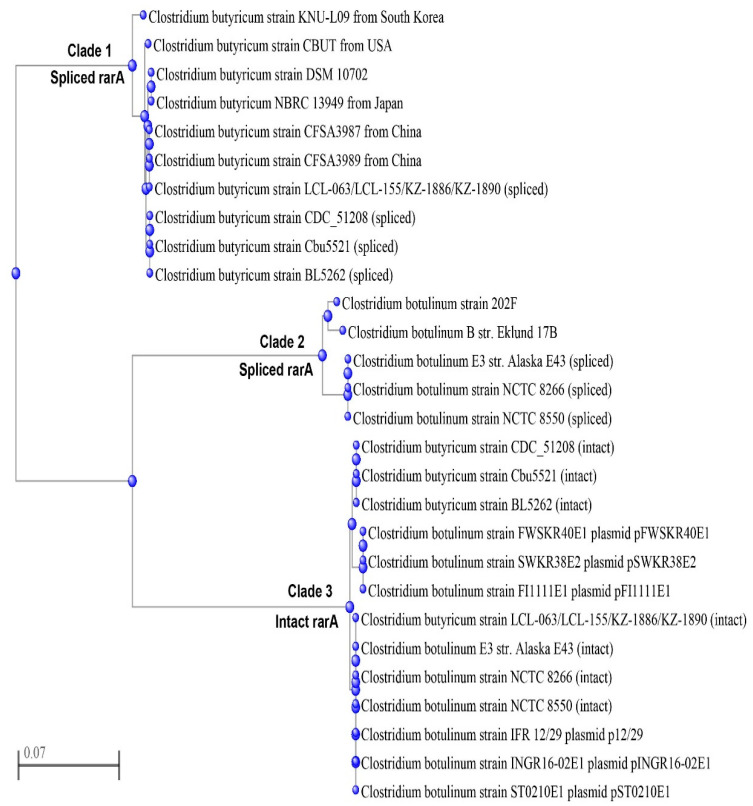
A fast minimum evolution tree of the intact and spliced *rarA* gene sequences from different *C. botulinum* and *C. butyricum* type E strains. Many *C. botulinum* or *C. butyricum* strains contain two copies of *rarA* genes, of which one is intact while the other one is split from the insertion of the *bont*/*e* complex genes. Clade 1 is a class of *rarA* gene homologous to the spliced (reconstructed) *rarA* gene of Chinese strains. Clade 2 includes the sequences homologous to the spliced *rarA* gene of *C. botulinum* E3 strain Alaska E43. Clade 3 is a class of highly homologous *rarA* genes including the intact *rarA* genes of Chinese *C. butyricum* type E strains.

A fast minimum evolution tree of the *rarA* gene sequences from different *C. botulinum* and *C. butyricum* type E strains shows that they fall into three clades (Figure 5).

In clade 1, the spliced (reconstructed) *rarA* gene of the Chinese *C. butyricum* type E strains shared 100% coverage and identity with an intact *rarA* gene of the non-neurotoxigenic *C. butyricum* strains CFSA 3987 and CFSA 3989, both from China. A 99.6% nucleotide identity level (five nucleotides difference) was observed with the spliced (reconstructed) *rarA* genes in the genomes of the neurotoxigenic *C. butyricum* type E strains CDC 51208, BL 5256 and Cbu 5521 (Figure 5).

Clade 2 included a class of spliced (reconstructed) *rarA* genes of the *C. botulinum* strains Alaska E 43, NCTC 8266 and NCTC 8550, which were similar to the intact *rarA* genes of *C. botulinum* type B and F strains Eklund 17 B and 202 F, respectively (Figure 5).

The observed difference between the spliced (reconstructed) *rarA* genes in clades 1 and 2 confirms that the insertion of the *bont*/*e* gene cluster occurred as separate events in the *C. butyricum* and *C. botulinum* species, in accordance with previous studies [17].

In clade 3, the intact *rarA* gene in the 27 kb insert region of the Chinese *C. butyricum* type E genomes was identical (100% coverage and identity) to the *rarA* gene sequences in the chromosome of the *C. botulinum* type E strains Alaska E 43, NCTC 8266 and NCTC 8550, and in the plasmids p12/29, pINGR16-02E1 and pST0210E1 of the *C. botulinum* type E strains IFR 12/29, INGR16-02E1 and ST0210E1k, respectively. One nucleotide difference (1234/1245 nucleotides, 100% coverage) was observed with the intact *rarA* gene of the neurotoxigenic *C. butyricum* type E strains CDC 51208, BL 5256, and Cbu 5521 (Figure 5).

The observation that the intact (inserted) *rarA* genes of all BoNT/E-producing *C. botulinum* and *C. butyricum* strains were almost identical to each other supports the hypothesis of a common source for genetic insert regions [17,20].

#### 2.3.3. *Transposase* Genes

**Figure 6:**
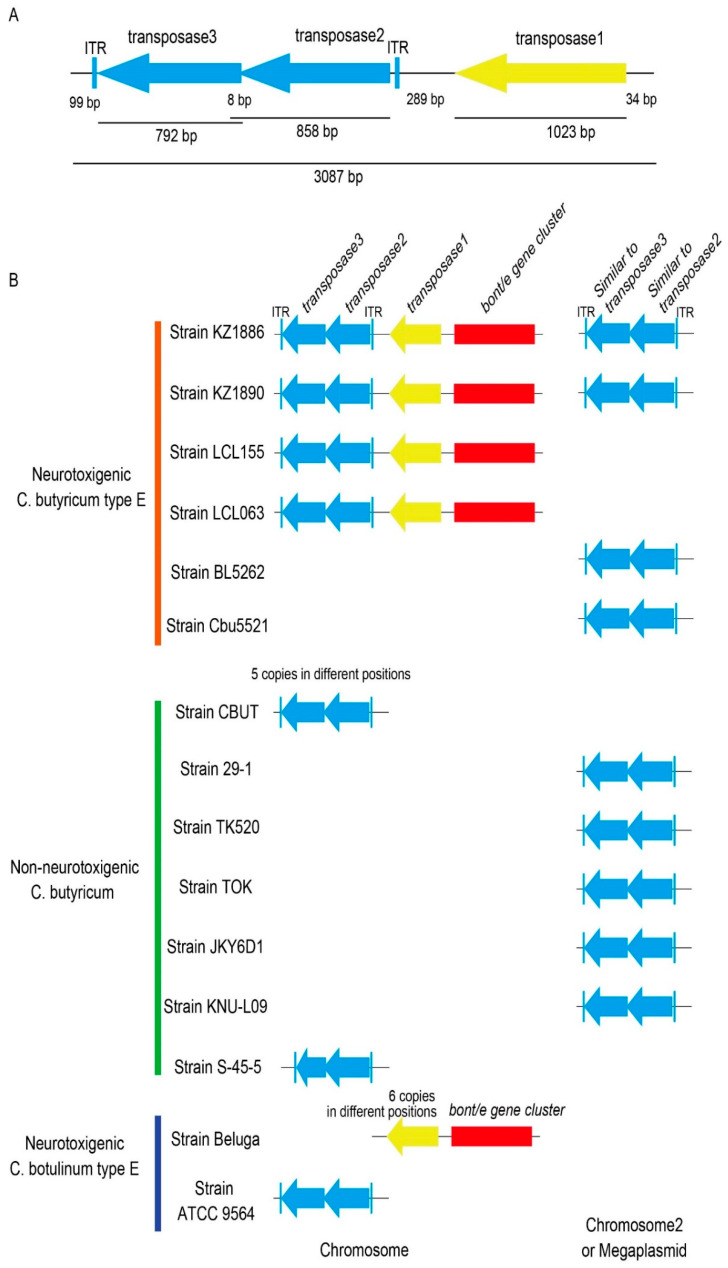
(**A**) Schematic diagram of the *transposase* genes showing the lengths and relative locations of the three transposase genes. (**B**) Transposase genes (type, position, and copy number) in strains of neurotoxigenic *C. butyricum* type E, non-neurotoxigenic *C. butyricum*, and neurotoxigenic *C. botulinum* type E. The blue arrows correspond to *transposase2* and *transposase3*, which make up part of the putative TE.

The genome of the *C. butyricum* type E strain KZ 1886 contained three *transposase* genes upstream of the *bont*/*E* gene cluster (NODE_1_length_307347) (Figure 6): *transposase1* (PIBAPCPA_01466, 1023 bp), *transposase2* (PIBAPCPA_01467, 858 bp), and *transposase3* (PIBAPCPA_01468, 792 bp). *Transposase2* and *transposase3* shared 8 bp.

*Transposase1* (340 aminoacids) belongs to the *IS6* transposase family (K18320) in the KEGG Orthology (KO), and *transposase2* (285 aminoacids) and *transposase3* (263 amino acids) belong to the *ISL3* transposase family (K07485) in the KO.

Two additional *transposase* genes (PIBAPCPA_00979, 858 bp; PIBAPCPA_00980, 792 bp) showing nucleotide similarities to *transposase2* and *transposase3*, respectively, were also identified in a different scaffold (NODE_67_length_5230) of the genome sequence of strain KZ 1886. The similarity level between PIBAPCPA_01467 and PIBAPCPA_00979 was 84.3% (723/858 nucleotides), whereas the similarity level between PIBAPCPA_01468 and PIBAPCPA_00980 was 80.8% (640/792 nucleotides) (Figure 6).

Likewise, the genome of strain KZ 1890 also included three *transposase* genes upstream of the *bont*/*E* gene cluster (NODE_419_length_3392): *transposase1* (HJKLNAAI_03406, 1023 bp), *transposase2* (HJKLNAAI_03407, 858 bp), and *transposase3* (HJKLNAAI_03408, 792 bp).

In addition, a *transposase* gene (HJKLNAAI_03980, 858 bp) similar to the *transposase2* gene HJKLNAAI_03407 (83.9% identity, 720/858 nucleotides) was present in a different scaffold (NODE_344_length_4393). Another *transposase* gene showing similarity to the *transposase3* gene (HJKLNAAI_03408) was divided into two different scaffolds, NODE_344_length_4393 and NODE_494_length_2643. After manual assembly of this gene, we located it adjacent to the *transposase* gene (HJKLNAAI_03980) and observed that its nucleotide sequence was highly similar (>99%) to that of the *transposase* gene PIBAPCPA_00980 of strain KZ 1886.

Finally, the *transposase1*, *transposase2* and *transposase3* genes were also identified in the genomes of strains LCL 155 and LCL 063 upstream of the *bont*/*e* gene cluster, as in the strains KZ 1886 and KZ 1890. However, no similar *transposase* genes were found elsewhere in the genomes of those strains (Figure 6).

We then screened the genome sequences of the *C. butyricum* type E strains CDC 51208, BL 5262 and Cbu 5521 for the presence of *transposase* genes similar to those identified in the genomes of the Chinese *C. butyricum* type E strains.

The US *C. butyricum* type E strain CDC 51208 genome sequence contained no regions with sequences similar to those of any of the three *transposase* genes of the Chinese strains.

By examining the genome sequence of the Italian *C. butyricum* type E strain BL 5262, a 1627 bp genetic region comprising two *transposase* genes similar to *transposase2* and *transposase3* was detected in the megaplasmid sequence (contig60, 757,653 bp, NZ_ACOM01000001.1), spanning from nucleotides 582337 to 583963. This region shared 99% and 87% similarity levels with the scaffolds (NODE_67_length_5230) and (NODE_1_length_307347) of the genome sequence of strain KZ 1886, which likely corresponded to megaplasmid and chromosomal sequences, respectively. A genetic region identical to that of strain BL 5262 was also found in the genome of the other Italian strain Cbu 5521 (gcontig_1106103650376, 139,815 bp, ABDT01000049.2) spanning from nucleotide 98687 to 100313 (Figure 6).

Furthermore, a similar 1642 bp region containing the *transposase2* and *transposase3* genes was identified in the megaplasmid (also called chromosome 2) sequence of six non-neurotoxigenic *C. butyricum* strains (CBUT, 29-1, TK520, TOA, JKY6D1 and KNU-L09) (Figure 6). Those regions shared 99% and 87% similarity with the homologous regions in the megaplasmid and chromosome of strain KZ 1886, respectively. The non-neurotoxigenic *C. butyricum* strain CBUT contained five additional copies of the *transposase2* and *transposase3* genetic region, in addition to the copy in the megaplasmid (KDJ93_18275 and KDJ93_18280), dispersed in the chromosome (KDJ93_03675 and KDJ93_03680, KDJ93_03695 and KDJ93_03700, KDJ93_09385 and KDJ93_09390, KDJ93_15250 and KDJ93_15255, and KDJ93_15270 and KDJ93_15275). The six copies of the *transposase2* and *transposase3* genetic region (1642 bp) were 100% identical to each other. Strain S-45-5 possessed an intact *transposase2* gene (DRB99_14020) and an incomplete *transposase3* gene (DRB99_14030) in the chromosome (3.81 Mb) (Figure 6).

Concerning the *C. botulinum* species, the region comprising the *transposase2* and *transposase3* genes was only identified in the chromosome of the *C. botulinum* type E strain ATCC 9564, where it was not linked to the *bont*/*e* gene cluster. These two *transposase* genes (ACP51_01210, ACP51_01215) were 100% identical to the *transposase2* and *transposase3* genes upstream of the *bont*/*e* gene cluster in the chromosome of the Chinese neurotoxigenic *C. butyricum* type E strains.

The chromosomal and plasmid regions containing the *transposase2* and *transposase3* genes in the Chinese *C. butyricum* type E strains genome sequences, and those identified in all other analyzed *C. butyricum* genomes and *C. botulinum* ATCC 9564 genome, were flanked by inverted terminal repeats (ITRs) similar to each other with only a few nucleotide mutations (Figure S2, Supplementary Materials).

Thus, these findings clearly indicate that the various genetic regions containing the *transposase2* and *transposase3* genes and surrounded by almost identical ITRs are copies of the same putative TE.

As noted earlier, all four Chinese *C. butyricum* type E strains analyzed here contained a chromosomal copy of the putative TE which, along with a *transposase1* gene, was located upstream of the *bont*/*e* gene cluster. Only the soil strains KZ 1886 and KZ 1890 possessed a second copy of the putative TE within the megaplasmid (Figure 6).

Regarding the *transposase1* gene, six copies of a gene showing varying similarity levels (99.32–100%) to the one flanking the *bont*/*e* gene cluster of the Chinese *C. butyricum* type E strains were only identified in the genome of the *C. botulinum* type E strain Beluga, all in CLO.Contig141 (ACSC01000002.1, 3.86 Mb); one of these genes was located upstream of the *bont*/*e* gene cluster [17] (Figure 6).

## 3. Discussion

The four neurotoxigenic *C. butyricum* type E strains from China that were sequenced in this study are among the rare BoNT-producing strains belonging to species other than *C. botulinum*. Together with the neurotoxigenic *C. butyricum* type E strains isolated to date in Italy, India, Japan, the United Kingdom and the United States [4,5,6,9,10,11,25,26,27], along with non-neurotoxigenic *C. butyricum* strains, they form phylogenetic clostridia Group VI.

The comparative analysis of the genome sequences obtained in this study and those available for another three neurotoxigenic *C. butyricum* type E strains revealed a certain degree of diversity in the BoNT-producing clostridia of Group VI, similar to previous reports for other phylogenetic clostridia groups [28].

The early identification of BoNT/E-producing *C. butyricum* strains, along with BoNT/F-producing *C. barati* strains during the 1980s, raised the possibility that *bont*-encoding genes might be subject to lateral transfer [2].

Over time, *bont* gene mobility has become increasingly apparent [29], with the latest indirect evidence provided by the findings of putative *bont*-related genes even in the genome of non-clostridial genera, including Weissella, Enterococcus, Chryseobacterium, Mycobacterium and Actinobacteria [30].

Although the only BoNT producers that have been identified so far are clostridia strains, the possible *bont* gene transfer to clostridial and even non-clostridial strains is of concern for both public health and biodefense reasons, as BoNT is one of the most poisonous natural substances to humans and animals [31]. Thus, understanding all potential mechanisms underlying *bont* gene mobilization is important.

From this perspective, WGS analysis has proved to be a powerful tool for predicting *bont* gene mobility. Regarding the *bont*/*e* gene, the results of a WGS analysis of different BoNT/E-producing strains previously suggested that it might be mobile through transposition, even though TEs close to the *bont*/*e* gene were never reported, and/or plasmid conjugation [17,20].

Here, for the first time we provide evidence that the *bont*/*e* gene cluster of all analyzed Chinese *C. butyricum* type E strains is preceded at the 5′ end by a putative TE consisting of a genetic region that includes two intact ISL3 *transposase* genes and flanked at both ends by ITRs. Our WGS data also showed an additional IS6 *transposase* gene between the putative TE and the *bont*/*e* gene cluster.

By searching the available genome sequences, a similar putative TE was only found in the genome of the *C. botulinum* type E strain ATCC 9564. The genome of another *C. botulinum* type E strain Beluga contained an IS6 transposase gene upstream of the *bont*/*e* gene cluster. These findings reinforce the hypothesis that the *bont*/*e* gene of the *C. butyricum* type E strains might have originated from *C. botulinum* type E strains through transposition.

TEs, also known as transposons or “jumping genes”, can pick up genomic sequences and by using transposases can allow intracellular and inter-cellular movements of DNA by homologous recombination between similar or identical TEs [32]. The IS6 transposase might be involved in facilitating the integration of TEs at insertion sites.

We identified a second copy of the putative TE in different scaffolds, likely part of the megaplasmid sequence, of the genomes of both soil strains (KZ 1886 and KZ 1890); thus, we hypothesize that, at least in those environmental strains, under certain favorable conditions, the *bont*/*e* gene cluster might pass from the chromosome to the megaplasmid and vice versa.

The single copy of the putative TE that was also found within the megaplasmid of several of *C. butyricum* strains, including the Italian BoNT/E-producing ones, might function as a potential acceptor of the *bont*/*E* gene cluster from other strains. However, no *bont*/*e*-bearing megaplasmids have yet been identified *in C. butyricum* strains and curing the megaplasmid of two Italian neurotoxigenic *C. butyricum* type E strains did not affect the levels of *bont*/*e* production [33].

TEs can also be transmitted to other bacteria by the transfer of plasmid DNA [32] but whether the megaplasmids of the *C. butyricum* strains are conjugable remains unknown.

Finally, the fact that the US strain of neurotoxigenic *C. butyricum* type E CDC 51208 did not carry any copies of the putative TE that we identified in the Chinese *C. butyricum* type E strains may indicate that the strain has lost it, possibly increasing the stability of the *bont*/*e* gene in its genome.Currently, two evolutionary interpretations exist concerning the significance of *bont* gene acquisition and maintenance in clostridia strains. According to the classic view, *bont* genes confer an advantage to their clostridia hosts because BoNT production ultimately provides a suitable anaerobic substrate for the spread of clostridia [1]. Recently, a rare chromosomal integration of *bont*-encoding plasmids has been observed in different clostridia species and has been suggested to facilitate the stable retention of the *bont* gene in clostridia hosts [34].

By contrast, *bont* genes could also be regarded as “selfish genes” that tend to propagate themselves in suitable host bacteria, thus persisting over time; in this case, BoNT production would be independent from bacteria proliferation [35].

The findings of our study favor the former hypothesis: in fact, if on one side the identification of a putative TE close to the *bont*/*e* gene cluster would support its mobilization within and among bacterial genomes, the presence of putative TE in a few strains of only two clostridia species, *C. butyricum* and *C. botulinum* type E, greatly reduces its transfer potential as a selfish gene.

Thus, our results might provide an explanation for the rare isolation of neurotoxigenic *C. butyricum* type E.

## 4. Materials and Methods

### 4.1. Bacterial Strains

The neurotoxigenic *C. butyricum* type E strains LCL 063, LCL 155, KZ 1886 and KZ 1890 were a kind gift from Professor Shinichi Nakamura of Kanazawa University to Dr Paolo Aureli of the Istituto Superiore di Sanità. Strains were maintained at −80 °C in cryogenic microbank vials (Prolab Diagnostics, Round Rock, TX, USA). Prior to DNA extraction, stock cultures of the strains were transferred to egg yolk agar (EYA) plates and checked for purity after 48 h of incubation at 37 °C under anaerobic conditions (Gas Pack anaerobic jars and AnaeroGen atmosphere generation). The four neurotoxigenic *C. butyricum* type E strains were confirmed to be positive for the *bont*/*e* gene by PCR, as previously described [36].

### 4.2. DNA Extraction

For DNA extraction, a single colony of each *C. butyricum* type E strain was selected from EYA plates, inoculated in 9 mL of tryptone–peptone–soy–yeast extract (TPGY) broth, and incubated overnight at 37 °C under anaerobic conditions. Genomic DNA was prepared from 1.5 mL of the overnight TPGY broth cultures of the four strains using the DNEasy Tissue kit (Qiagen, Hilden, Germany) following the manufacturer’s instructions, including a cell lysis step as recommended for Gram-positive bacteria. The quality and quantity of the isolated DNA were estimated using agarose gel electrophoresis and an ultraviolet spectrophotometer.

### 4.3. Genome Sequencing, Assembly and Annotation

The genomes of strains KZ 1886, KZ 1890, LCL 063 and LCL 155 were sequenced using an Illumina MiSeq (Illumina, San Diego, CA, USA). Softwares Velvet1.2.10 (EMBL-EBI, Cambridge, United Kingdom) and SSPACE-STANDARD-3.0 (Leiden University, Leiden, The Netherlands) were used to assemble the preprocessed reads, and GapFiller v2.1.1 (University of Udine, Udine, Italy) was used to further fill the gaps. Genome annotation was performed using Prokka1.14.6 (Victorian Bioinformatics Consortium, Carlton, Australia) [37].

### 4.4. Comparative Genomics

The draft genome sequences generated in this study for each of the four neurotoxigenic *C. butyricum* type E5 strains from China were compared with those publicly available for three neurotoxigenic *C. butyricum* type E4 strains: the Italian strains BL 5262 (accession No. NZ_ACOM00000000.1) and Cbu 5521 (accession No. NZ_ABDT00000000.1) and the US strain CDC 51208 (accession Nos. NZ_CP013239.1; NZ_CP013240.1 and NZ_CP013238.1). The genome sequences of the reference (non-neurotoxigenic) *C. butyricum* strain KNU-L09 (accession Nos NZ_CP013252.1 and NZ_CP013489.1), CBUT (accession Nos NZ_CP073277.1 and NZ_CP073278.1), CFSA3987 (accession Nos NZ_CP033247.1 and NZ_CP033246.1), CFSA3989 (accession Nos NZ_CP033249.1 and NZ_CP033248.1), and *C. botulinum* type E strain Alaska E43 (accession No. NZ_CP001078.1) were included in the comparative analyses as representatives of the *C. butyricum* and *C. botulinum* species. Single nucleotide polymorphisms (SNPs) between all isolated strains were identified using the kSNP3 software (Lawrence Livermore National Laboratory, Livermore, CA, USA) [38] with the default parameters. The SNPs were used to construct the phylogenetic tree using the maximum likelihood method with RAxML v8.1.6 (Karlsruhe Institute of Technology, Karlsruhe, Germany). A phylogenetic analysis was performed using the maximum likelihood method with RAxML v8.1.6 [39]. The megaplasmid sequences were interfered with by the available closed genome sequence of the neurotoxigenic *C. butyricum* strain CDC-51208 (820 kb plasmid, pNPD4_2, accession No. CP013238.1).

### 4.5. Comparative Analyses of the Bont/e Gene Clusters and Adjacent Regions

The contigs obtained through the assembly process for the four neurotoxigenic *C. butyricum* type E strains were searched through BLAST for the *bont*/*e* gene cluster and adjacent genes. A BLAST search with the NCBI nucleotide collection (nr/nt) or whole-genome shotgun contigs (wgs) database was used to identify the homologous *bont*/*e*, *rarA* and *transposase* genes in other strains.

### 4.6. Data Deposition

The obtained draft genome sequences were deposited in GenBank with accession Nos: JAJDMT000000000 for strain LCL-155, JAJDMU000000000 for strain LCL-063, JAJDMV000000000 for strain KZ 1890, and JAJDMW000000000 for strain KZ 1886.

## Figures and Tables

**Table 1:** General features of the *C. butyricum* type E genome sequences.

Strain	Genome Size (bp)	G + C Content (%)	No. of Contigs	No. of Coding Sequences	No. of tRNA Genes
KZ 1886	4,633,810	28.58%	152	4232	91
KZ 1890	4,575,226	28.78%	986	4179	86
LCL-155	4,577,534	28.90%	227	4043	91
LCL-063	4,523,830	28.70%	473	4091	86

## Data Availability

The genome sequences generated in this study are available under Genbank accession Nos. JAJDMT000000000; JAJDMU000000000; JAJDMV000000000; and JAJDMW000000000. The sequences can be found at: https://www.ncbi.nlm.nih.gov/bioproject/PRJNA770765, accessed on 5 December 2021.

## Supplementary Materials

The following supporting information can be downloaded at https://www.mdpi.com/article/10.3390/ijms23020906/s1.
