# Supplementary material for: Novel Putative Transposable Element Associated with the Subtype E5 Botulinum Toxin Gene Cluster of Neurotoxigenic Clostridium butyricum Type E Strains from China
Source: Int J Mol Sci. 2022 Jan 14;23(2):906. doi: 10.3390/ijms23020906 (PMC8776182; doi:10.3390/ijms23020906)
Supplement: Supplementary file 1 [file ijms-23-00906-s001.zip › ijms-1483778-supplementary.pdf]

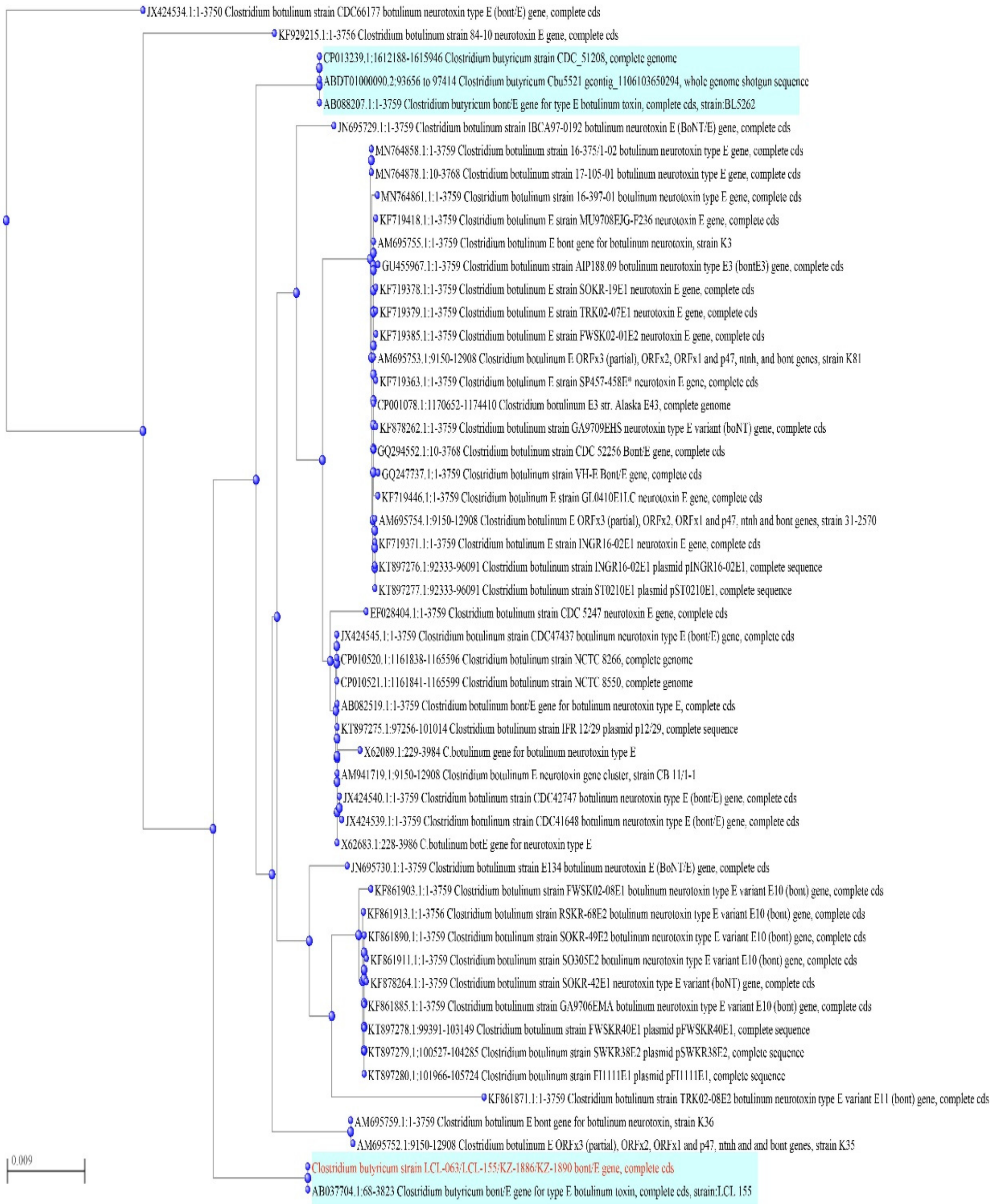

Figure S1. Fast minimum evolution trees of the *bont/e* gene sequences of different BoNT/E-producing clostridia strains. The dendrogram shows the variation in the *bont/e* gene. The analysis included the *bont/e* gene from the Chinese strains (red front) and all 51 highly homologous (> 99% coverage) sequences in the nucleotide collection (nr/nt) in the NCBI database. Six highly homologous *bont/e* genes are from *C. butyricum* strains (marked with a blue background).

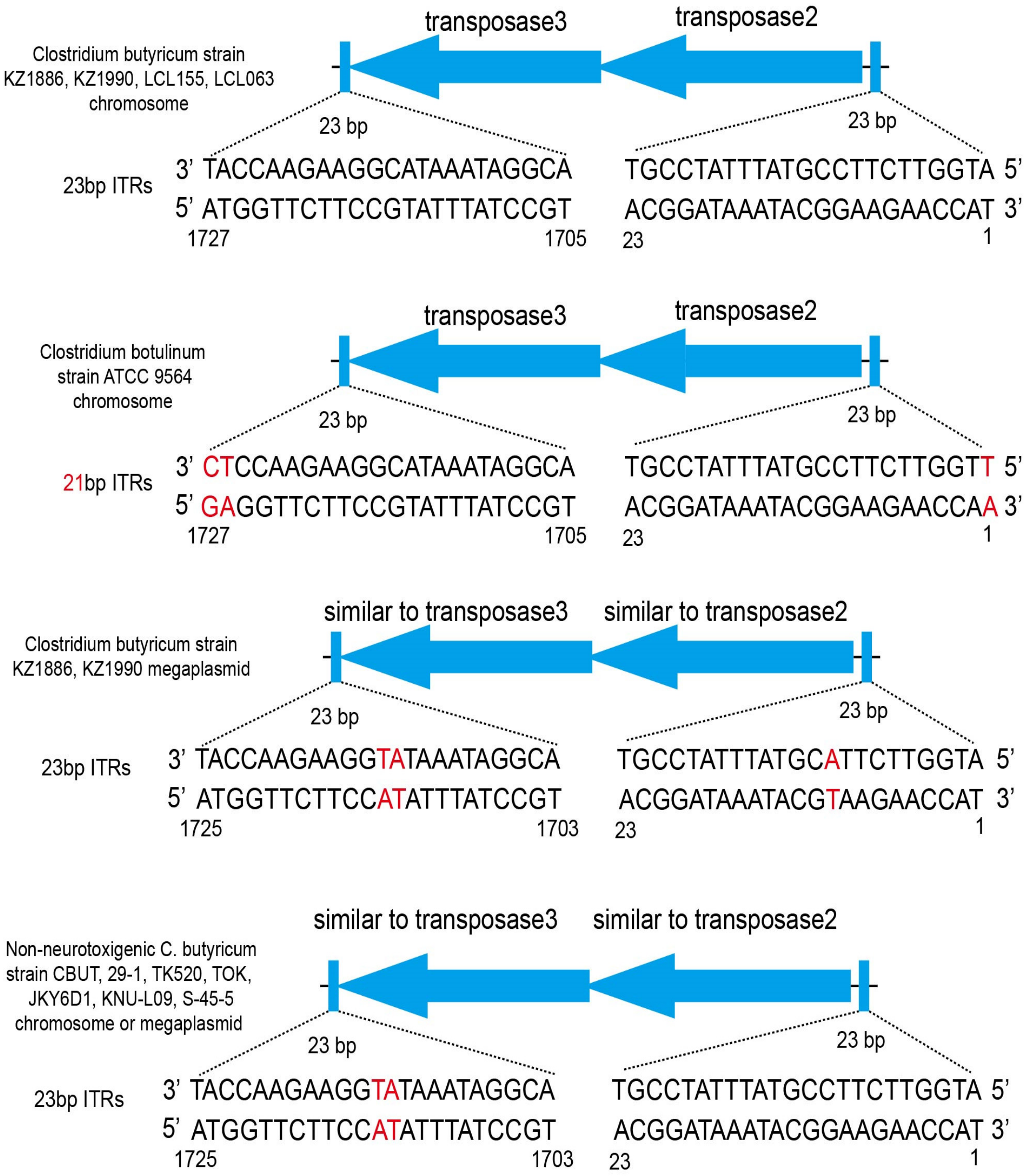

Figure S2. Comparison of the TEs. The nucleotide differences in the ITRs are marked in red.
